# French Endocrine Society Guidance on endocrine side effects of immunotherapy

**DOI:** 10.1530/ERC-18-0320

**Published:** 2018-10-08

**Authors:** F Castinetti, F Albarel, F Archambeaud, J Bertherat, B Bouillet, P Buffier, C Briet, B Cariou, Ph Caron, O Chabre, Ph Chanson, C Cortet, C Do Cao, D Drui, M Haissaguerre, S Hescot, F Illouz, E Kuhn, N Lahlou, E Merlen, V Raverot, S Smati, B Verges, F Borson-Chazot

**Affiliations:** 1 Aix-Marseille Université, Institut National de la Santé et de la Recherche Médicale (INSERM), U1251, Marseille Medical Genetics (MMG), and Department of Endocrinology, Assistance Publique-Hôpitaux de Marseille (AP-HM), Hôpital de la Conception, Centre de Référence des Maladies Rares de l’Hypophyse HYPO, Marseille, France; 2 Service de Médecine Interne B – Endocrinologie, Limoges Cedex, France; 3 Hôpital Cochin, Service d’Endocrinologie et Maladies Métaboliques, Paris Cedex 14, France; 4 CHU Dijon, Hôpital François Mitterrand, Service d’Endocrinologie, Diabétologie, Maladies Métaboliques, Dijon Cedex, France; 5 Unité INSERM LNC-UMR 1231, Université de Bourgogne, Dijon, France; 6 Institut MITOVASC, INSERM U1083, Angers University, Department of Endocrinology, Diabetology and Nutrition, University Medical Center, Angers, France; 7 Department of Endocrinology, L’Institut du Thorax, CHU Nantes, Nantes, France; 8 CHU de Toulouse – Hôpital Larrey – Service d’Endocrinologie – Maladies métaboliques – Nutrition, TSA 30030, Toulouse Cedex 9, France; 9 CHU de Grenoble – Hôpital Albert Michallon, Service d’Endocrinologie-Diabétologie-Nutrition, Grenoble Cedex 9, France; 10 Assistance Publique-Hôpitaux de Paris (AP-HP), Hôpital de Bicêtre, Service d’Endocrinologie et des Maladies de la Reproduction, et UMR S-1185 Faculté de Médecine Paris-Sud, University of Paris-Saclay, Le Kremlin-Bicêtre, France; 11 CHRU de Lille – Hopital Huriez, Service d’Endocrinologie, Lille Cedex, France; 12 CHU de Bordeaux – Hôpital du Haut Lévêque, Service d’Endocrinologie-Diabétologie et Maladies Métaboliques, Pessac Cedex, France; 13 Institut Curie, Oncologie Endocrinienne, Saint Cloud, France; 14 Department of Endocrinology, Diabetes and Nutrition, Reference Centre of Rare Thyroid Disease, Hospital of Angers, Angers Cedex 09, France; 15 Département d’Hormonologie Spécialisée, BPR-AS, Pannes, France; 16 Hospices Civils de Lyon, Laboratoire d’Hormonologie, Service de Biochimie et Biologie Moléculaire, Groupement Hospitalier Est, Lyon, France; 17 Hospices Civils de Lyon, Fédération d’Endocrinologie, Université Claude Bernard Lyon 1, Lyon, France

**Keywords:** immune checkpoint inhibitor, CTLA-4, PD-1, PD-L1, hypothyroidism, thyrotoxicosis, hypophysitis, diabetes, adrenal insufficiency

## Abstract

The management of cancer patients has changed due to the considerably more frequent use of immune checkpoint inhibitors (ICPIs). However, the use of ICPI has a risk of side effects, particularly endocrine toxicity. Since the indications for ICPI are constantly expanding due to their efficacy, it is important that endocrinologists and oncologists know how to look for this type of toxicity and how to treat it when it arises. In view of this, the French Endocrine Society initiated the formulation of a consensus document on ICPI-related endocrine toxicity. In this paper, we will introduce data on the general pathophysiology of endocrine toxicity, and we will then outline expert opinion focusing primarily on methods for screening, management and monitoring for endocrine side effects in patients treated by ICPI. We will then look in turn at endocrinopathies that are induced by ICPI including dysthyroidism, hypophysitis, primary adrenal insufficiency and fulminant diabetes. In each chapter, expert opinion will be given on the diagnosis, management and monitoring for each complication. These expert opinions will also discuss the methodology for categorizing these side effects in oncology using ‘common terminology criteria for adverse events’ (CTCAE) and the difficulties in applying this to endocrine side effects in the case of these anti-cancer therapies. This is shown in particular by certain recommendations that are used for other side effects (high-dose corticosteroids, contraindicated in ICPI for example) and that cannot be considered as appropriate in the management of endocrine toxicity, as it usually does not require ICPI withdrawal or high-dose glucocorticoid intake.

## Introduction and pathophysiology

The management of cancer has changed profoundly over the last 20 years, with the development of new approaches, particularly those based on the understanding of mechanisms underlying the immune response to neoplastic cells. The concept of immunotherapy (immune checkpoint inhibitors (ICPIs)) has given rise to many clinical trials that have shown the anti-tumoral efficacy of these molecules in indications as varied as melanoma and small-cell lung cancer. This has led to the use of ICPI now becoming widespread. The use of these new agents necessitates careful monitoring since they may cause numerous side effects, including the risk of developing endocrinopathies. However, despite universal recommendations for the management of side effects of these treatments (Haanen *et al.* 2017), there have been to date no recommendations from specialist societies on the management of endocrinopathies or diabetes induced by ICPI, with the exception of the management of acute complications (Higham *et al.* 2018). In 2017, the French Endocrine Society initiated work to summarize the current state of knowledge on the diagnosis and treatment of these induced endocrinopathies. Expert endocrinologists met three times between October 2017 and April 2018, and formulated an expert opinion based on an exhaustive literature review (using PubMed) with the search terms ‘ICPI, CTLA-4, PD-1, PD-L1, diabetes, hypophysitis, thyroiditis, adrenal insufficiency’, over the period 1990–2018. Feedback on the consensus document was then received from forty expert endocrinologists and oncologists, and it was then presented at the French Endocrine Society conference (Nancy, France, 2018) (Castinetti & Borson-Chazot 2018).

**Figure 1:**
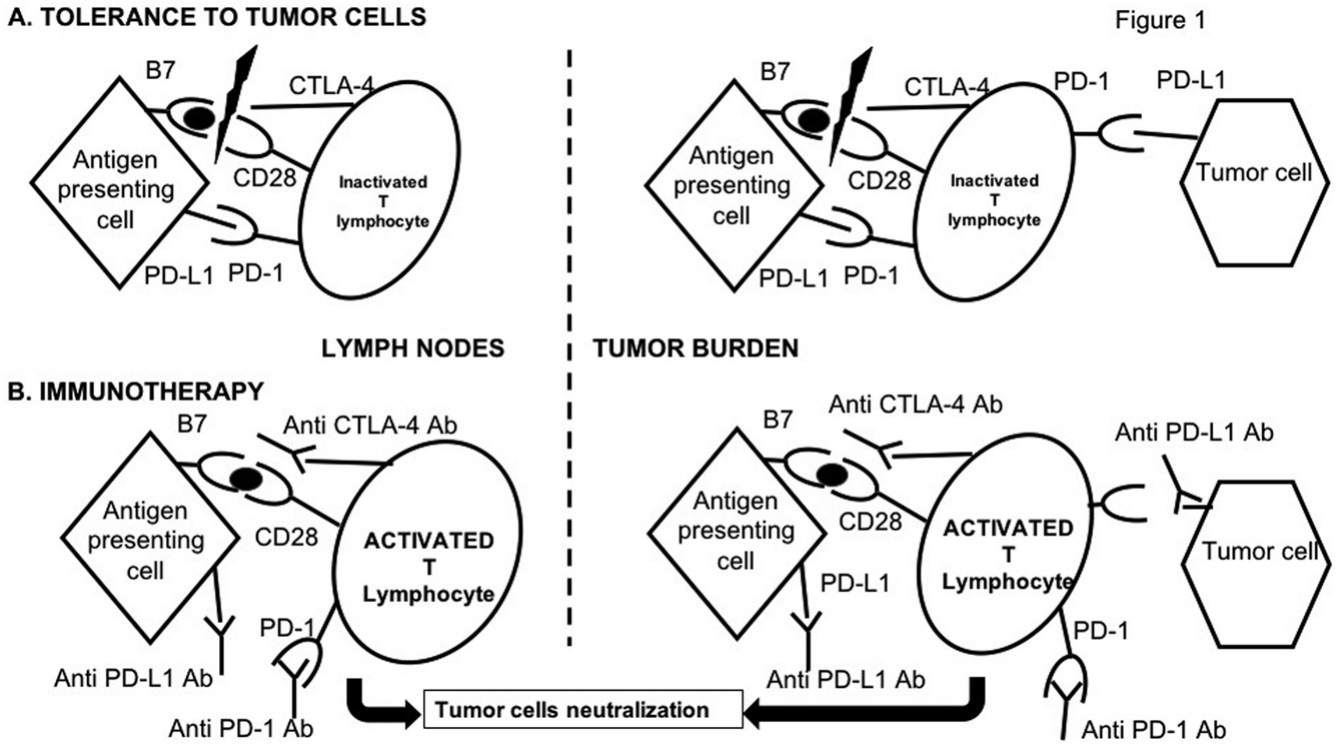
ICPI mechanisms. (A) The principal pathway of co-stimulation for activation of naïve T lymphocytes is the CD28/B7 pathway, consisting of an activating signal for T lymphocytes following binding of CD28 to B7. CTLA-4 can block this stimulatory pathway. Other inhibitory signals induced by binding of PD-1/PD-L1 occur in the lymph nodes and at the tumor site. (B) The principal treatments currently used are based on inhibition of CTLA-4 and/or of the PD-1/PD-L1 pair. This inhibition which results in prolonged activation of T lymphocytes directed against tumoral neoantigens, aims to neutralize tumor cells.

The role of ‘immune checkpoint’ proteins is to modulate the non-adaptive immune response, in particular, immune responses directed against self-antigens. These immune checkpoint molecules are necessary to regulate the immune response, both its activation and inhibition. Cancerous cells are capable of modifying the expression or effect of these co-stimulatory/co-inhibitory pathways (CTLA-4, PD-1, PD-L1) to avoid lymphocyte activation and to favor tolerance of the tumor cells. The objective of immunotherapies is thus to block molecules that have an inhibitory effect to thus allow reactivation of the immune response and favor destruction of the tumor cells, as shown in Fig. 1. For instance, PD-1 receptors are part of the immunoglobulin (Ig) superfamily and are expressed on the surface of activated T lymphocytes, B lymphocytes and monocytes. Ligands for PD-1 (L1 and L2) are present on the surface of antigen-presenting cells, non-lymphoid cells such as beta cells in islets of Langerhans, endothelial cells, cardiomyocytes and cancerous cells (Bour-Jordan *et al.* 2011). Binding of PD-1–PD-L1 inhibits the activation and proliferation of activated T lymphocytes. Binding of PD-1/PD-L2 decreases the production of pro-inflammatory cytokines (IL-2, IFN gamma) (Butte *et al.* 2008). Anti-PD-1 or anti-PD-L1 antibodies block this pathway and thus allow stimulation of an immune response directed against the tumor.

However, the mechanism of action underlying ICPI is also the origin of the immune side effects that can affect various organs. Side effects are most often light to moderate in severity, but 0.5–13% of patients present with grade 3–4 side effects forcing treatment to be stopped and in some cases necessitating treatment with immunosuppressive drugs (Khoja *et al.* 2017). The precise mechanism underlying these side effects is not completely understood (Postow & Hellmann 2018). For instance, for anti-CTLA-4, the inactivation of CTLA-4 appears to be the cause of auto-immune damage and cell death due to tissue infiltration by T lymphocytes (as described in animal models); it may lead to a loss of activity of T lymphocyte regulators, reducing the phenomenon of self-tolerance; it may increase the levels of pre-existing antibodies that are responsible for these immune effects (Ryder *et al.* 2014); it may also lead to cytotoxicity directed against self-antigens, which leads to release of new auto-antigens, which are themselves targets for T lymphocytes, increasing the immune reaction. It remains unclear why the endocrine effects, induced by these auto-immune mechanisms, are more frequently associated with the pituitary and thyroid. The rich vascularization of both these organs may make them more susceptible to contact with activated T lymphocytes; alternatively, direct expression of CTLA-4 in the pituitary or of PD-1/PD-L1 in the thyroid could explain why these organs are the most frequent targets through direct toxicity against the organ (Iwama *et al.* 2014, Caturegli *et al.* 2016).

Finally, it is likely that the endocrinopathy is correlated to the efficacy of ICPI. This is the case for hypophysitis, for example, where it was shown that the appearance of this side effect during ipilimumab treatment was correlated with a better anti-tumoral response in the melanoma (Faje *et al.* 2014, Faje 2016). However, these results could also be biased due to the longer exposure (and therefore greater risk of side effects) in patients that responded to ICPI. In practice, the mechanisms underlying the appearance of side effects are poorly understood and improving this would lead to a better understanding of the mechanism of action of ICPI while also providing predictive factors for the response to these drugs.

## 1. Initial testing and monitoring in the absence of endocrinopathy

**Figure 2:**
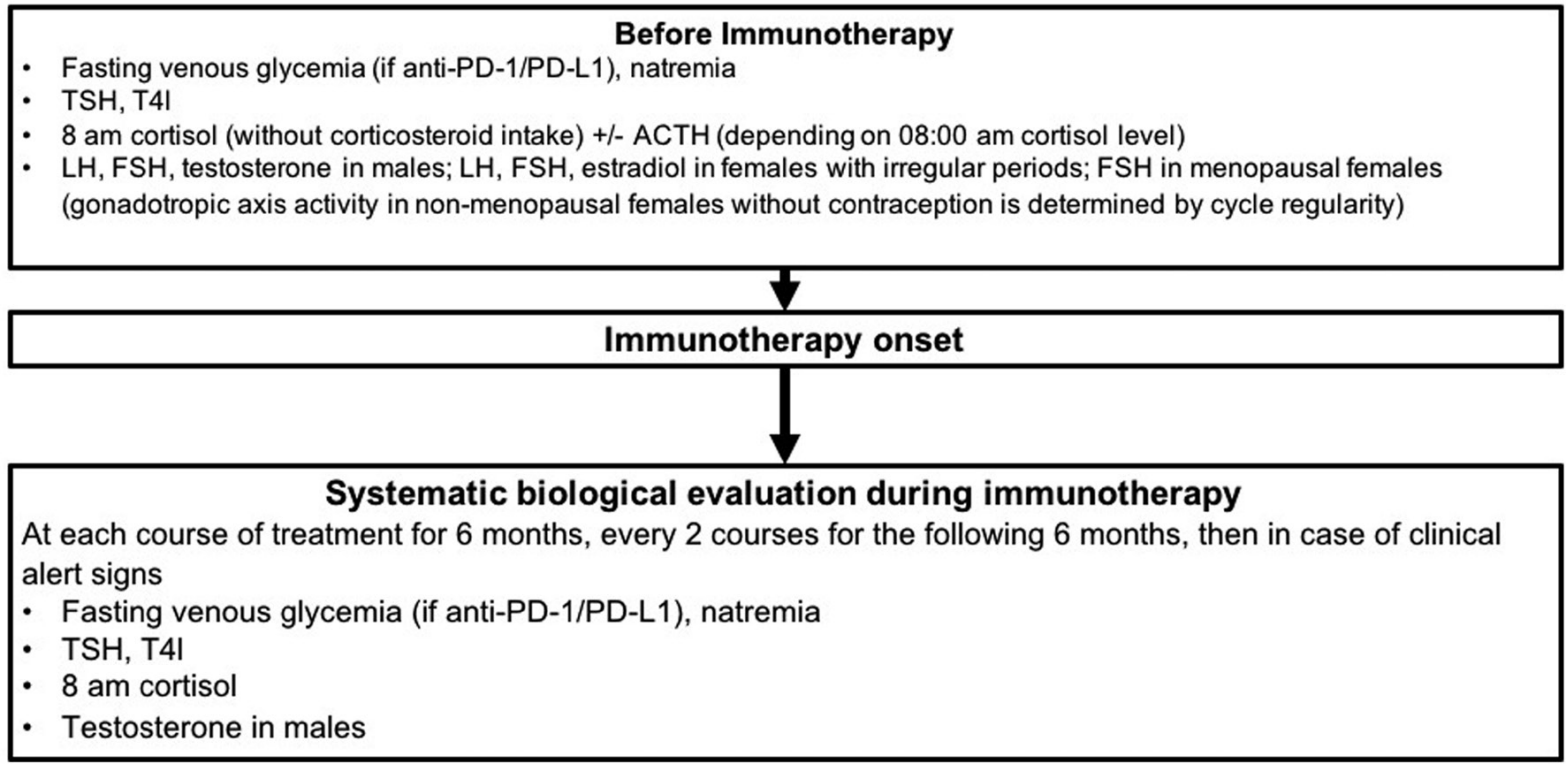
Screening and monitoring of endocrine toxicity in patients treated with ICPI.

Patients treated with ICPI are at risk of developing hypophysitis, thyroiditis and, to a lesser degree, diabetes or primary adrenal insufficiency (PAI). A hormonal screen before ICPI can confirm normal test results before treatment. It is also worthwhile testing over the course of treatment to follow the evolution of hormone levels during therapy (Castinetti *et al.* 2018). Of note, given the increase of use of anti-CTLA-4/anti-PD-1 association, we report here recommendations that can be applied whatever the ICPI used except for special cases (such as diabetes that has never been reported with anti-CTLA-4) (Fig. 2).

**R1a: Before ICPI onset, we recommend initial tests including fasting venous blood glucose (only with anti-PD-1/PD-L1), plasma sodium, TSH, free T4, 08:00 h cortisol (in the absence of corticosteroids), LH, FSH, testosterone in males and FSH in females post-menopause (the activity of the gonadotropin axis in women prior to menopause and not taking oral contraceptives being determined by the regularity of cycles). ACTH measurement should be performed in patients with 08:00 h cortisol <500 nmol/L. LH, FSH and estradiol should be measured in premenopausal women with irregular periods after exclusion of other non-iatrogenic etiologies**

The risk of appearance of an endocrinopathy is greater at the start of treatment, justifying closer monitoring over the first 6 months, followed by regular monitoring over the next 6 months and less frequently thereafter. Beyond this time, the risk of endocrinopathy becomes negligible though not completely absent. Endocrinopathy should therefore be considered only when there are clinical signs. Throughout ICPI treatment, both patient and oncologist should be aware of clinical signs suggestive of endocrine complications as detailed later.

**R1b: Over the course of ICPI treatment, we recommend systematic tests for fasting venous blood glucose (only with anti-PD-1/PD-L1), plasma sodium, TSH, free T4, 08:00 h cortisol and testosterone in males at each appointment during the first 6 months, and at every second appointment over the following 6 months, then on appearance of clinical signs. It is important that both the patient and oncologist be educated on recognizing clinical signs suggestive of endocrinopathies.**

## 2. Grading of ICPI-induced endocrinopathies

In general, grading of side effects of ICPI is based on the CTCAE on a scale of 1–5 (1 = light, 2 = moderate, 3 = severe, 4 = life threatening, 5 = death due to toxicity) (National Cancer Institute 2009). Briefly, the drug that is causing side effects should be theoretically stopped in cases of grade 2 side effects and resumed when symptoms/biochemical disturbances have regressed; methylprednisolone (0.5–1 mg/kg) should be started if symptoms persist for more than a week. In the case of grade 3 and 4 side effects, drug treatment should be stopped and the use of stronger doses of methylprednisolone (1–2 mg/kg) is recommended; for grade 4 side effects, resuming treatment with the drug is definitively contraindicated (Corsello *et al.* 2013, Champiat *et al.* 2016).

However, the case of endocrine or metabolic side effects is reasonably specific. As indicated in the following chapters, they are rarely grade 3 or 4 side effects. Any necessity for replacement therapy being considered a grade 2 side effect should not result in cessation of the anti-cancer drug: these endocrinopathies are easily treated using hormone replacement and in this case establishing equilibrium is rarely a problem. In cases of severe complications, the ICPI can be temporarily stopped. Even so, there is no known data showing that use of high-dose corticosteroid therapy changes the natural history of an ICPI-induced endocrinopathy.

**R2: Endocrinopathies induced by ICPI are most often easily equilibrated by hormone replacement (in the case of deficiency) or improved by symptomatic treatment in the case of hyperfunction. CTCAE should therefore be used with care and the development of an endocrinopathy does not justify contraindication of the anti-cancer therapy. In the case of severe side effects, ICPI can be temporarily suspended and later re-initiated with the agreement of the oncologist. The presence of an endocrinopathy induced by ICPI does not contraindicate the use of another anti-cancer therapy, including those of the same class.**

## 3. ICPI and immunoassay interference

Immunotherapies are based on antibodies that block control points in the anti-tumoral immune response (Hwang & Foote 2005). These are, in all cases, monoclonal antibodies and their common international denomination indicates their species of origin, before the final ‘mab’. Thus, the denomination ending in ‘o-mab’ represents murine antibodies; ending in ‘xi-mab’ for chimeric antibodies; in ‘zu-mab’ for humanized antibodies and finally in ‘u-mab’ for human antibodies. In the case where prescribed immunoassays rely on mouse monoclonal antibodies and where the therapeutic antibodies contain murine sequences, if the patients develop heterophile antibodies against the therapeutic antibody, analytical interference should be a concern. The interfering heterophile antibodies would be, depending on the case, HAMA (anti-mouse antibodies), HACA (anti-chimeric antibodies) or HAHA (anti-humanized antibody antibodies). It is therefore recommended, when immunoassays are prescribed for patients undergoing ICPI, to be certain of the exact nature of the therapeutic antibody (Lahlou & Raverot 2018). If the therapeutic antibody is likely to contain murine sequences then the presence of heterophile antibodies should be tested for and, if present, be neutralized (Scheen 2009, Marabelle & Gray 2015).

**R3: In the case of clinico-biochemical discordance, the type of therapeutic antibody administered needs to be known: murine (o), chimeric (xi), humanized (zu) or human (u). The probability of interference in an immunoassay using one or more antibodies developed in mice decreases from effectively 100% in the case of an o-mab, to likely 0% in the case of a u-mab.**

## 4. Induced dysthyroidism

Thyroid abnormalities constitute the most frequent endocrine side effects in the course of cancer ICPI. The underlying pathophysiological mechanisms of these thyroid dysfunctions remain incompletely understood. They consist mainly of silent inflammatory thyroiditis where the mechanism involves cytotoxicity of T lymphocytes. The role of natural killer cells has been implicated as these are increased in the case of thyroiditis occurring during anti-PD-1 treatment. The weak expression of CTLA-4 on circulating lymphocytes may explain the lower frequency of dysthyroidism over the course of treatment with anti-CTLA-4. Other mechanisms are also possible (e.g. potential roles of PD-1 and PD-L1 present in thyroid tissue) since thyroid dysfunction may not always be of auto-immune origin (de Filette *et al.* 2016, Delivanis *et al.* 2017, Osorio *et al.* 2017).

**Figure 3:**
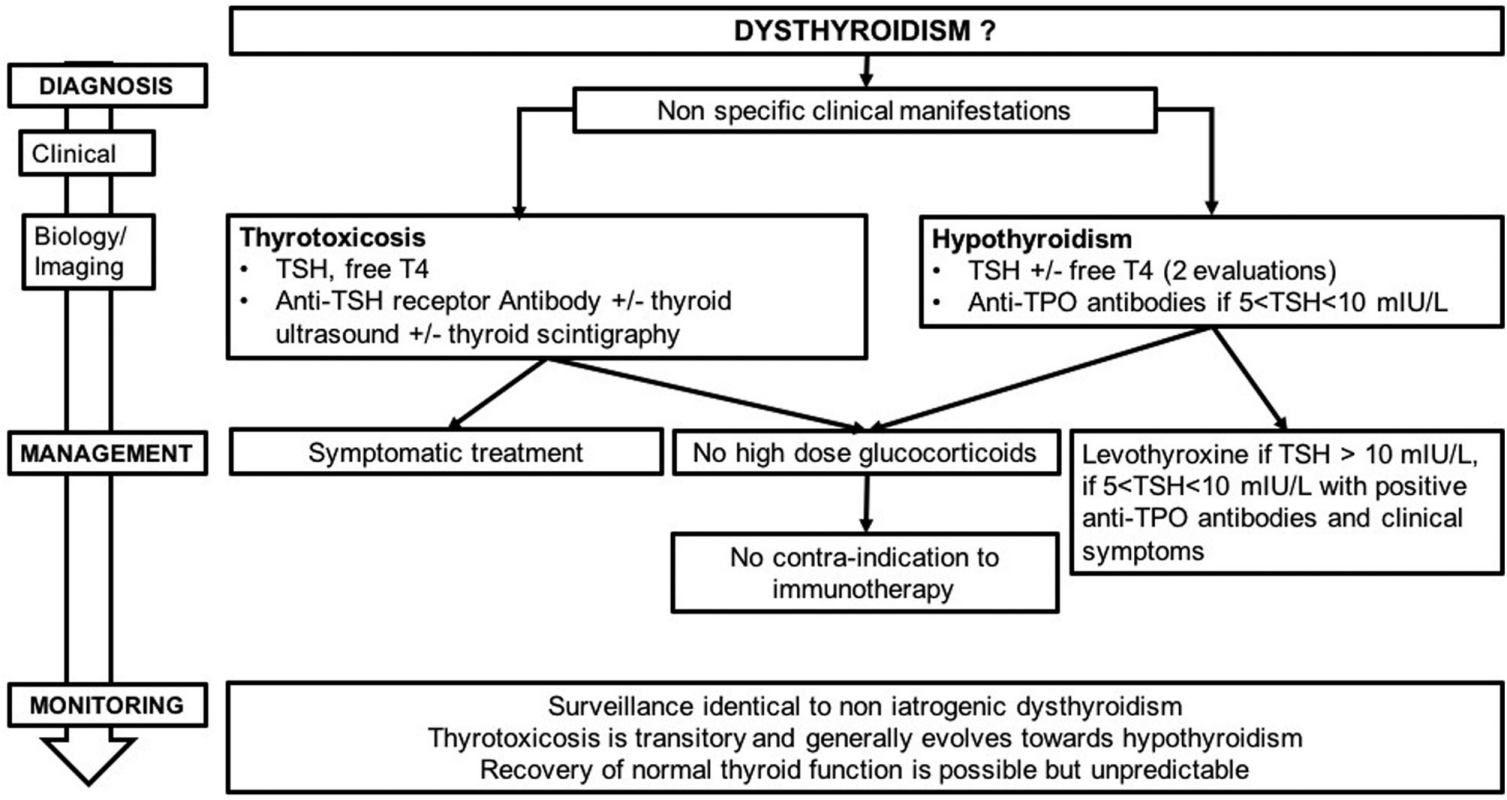
Management and monitoring of ICPI-induced dysthyroidism.

In a recent meta-analysis of 38 randomized trials, the risk of dysthyroidism was found to be higher in treatments combining anti-CTLA-4/anti-PD-1 than in monotherapy. The risk was also higher for anti-PD-1 treatments than for anti-CTLA-4, independent of tumor type (Larkin *et al.* 2015, Postow *et al.* 2015, Hodi *et al.* 2016, Weber *et al.* 2016, Long *et al.* 2017, Wolchok *et al.* 2017). This risk was dose dependent only in the case of anti-CTLA-4 treatment (particularly above a threshold of 10 mg/kg). The incidence of thyrotoxicosis varied from 3 to 16% and of hypothyroidism from 6 to 13%, notably as a function of therapeutic class, therapeutic sequence and whether mild or sub-clinical forms were taken into consideration. Incidence can reach 50% if mild forms are included (thyrotoxicosis 22% and hypothyroidism 28%, respectively) (Illouz *et al.* 2018) (Fig. 3).

### 4.1 Positive diagnosis

Recognizing the diagnosis and the severity of dysthyroidism in ICPI is comparable to dysthyroidism that is non-ICPI induced. Dysthyroidism is usually moderate or asymptomatic, most often classified as grade 1 or 2 in reported clinical trials (less than 1% of cases of severe dysthyroidism, classified grade 3 or 4) (Larkin *et al.* 2015, Ribas *et al.* 2015, Hodi *et al.* 2016, Reck *et al.* 2016, Weber *et al.* 2016,^ 2017^, Bellmunt *et al.* 2017, Gulley *et al.* 2017, Wolchok *et al.* 2017, Patel *et al.* 2018). Cases of thyroid storm or myxedema crisis have been rarely reported. As a result, the presence of dysthyroidism does not generally contraindicate ICPI treatment.

**R4.1a: The diagnosis of dysthyroidism during treatment by ICPI is based on assay of plasma TSH, since the clinical manifestations are non-specific.**

**R4.1b: The severity of thyrotoxicosis is assessed by its clinical impact and by increase in the level of free T4. The severity of hypothyroidism is assessed by clinical impact and increase in TSH levels.**

**R4.1c: The presence of a thyroid abnormality or pre-existing treated thyroid dysfunction is not a contraindication for ICPI. The development of thyroid dysfunction is not a contraindication for ICPI treatment. In the case of thyrotoxicosis or severe hypothyroidism, ICPI can be postponed, but in no case should be definitively contraindicated. In case of severe orbitopathy, ICPI should be stopped and re-initiated only after discussion on a case by case basis.**

### 4.2 Etiological diagnosis

Dysthyroidism arising during ICPI appears to be mainly due to inflammatory silent thyroiditis, that typically results in a phase of thyrotoxicosis followed by hypothyroidism (de Filette *et al.* 2016, Delivanis *et al.* 2017, Osorio *et al.* 2017). The picture can also less typically be of thyrotoxicosis that spontaneously resolves to euthyroidism or of the presence of hypothyroidism from the outset. The appearance of Graves’ disease, without associated orbitopathy, has also been reported in two cases. Conversely, some cases of orbitopathy without thyroid abnormality have been described in patients treated with ipilimumab and nivolumab (Min *et al.* 2011, McElnea *et al.* 2014, Campredon *et al.* 2018). In the case of hypothyroidism, the presence of anti-thyroid antibodies has not been consistently shown at the time of diagnosis (de Filette *et al.* 2016, Delivanis *et al.* 2017, Morganstein *et al.* 2017, Osorio *et al.* 2017): testing for these is recommended for the diagnosis of lymphocytic thyroiditis.

**R4.2a: Silent inflammatory thyroiditis is the most frequent etiological diagnosis linked to ICPI.**

- **In cases of thyrotoxicosis, assay for anti-TSH receptor antibodies, thyroid scintigraphy and Doppler ultrasound can be performed in case of uncertainty of diagnosis between iatrogenic thyroiditis and other diagnoses of hyperthyroidism, particularly in the case of severe thyrotoxicosis.**
- **In cases of hypothyroidism with an increase in TSH <10 mIU/L, assay for anti-TPO antibodies can help in the decision to begin supplementation with levothyroxine. This assay is less useful, in the treatment plan, when TSH concentrations are 10 mIU/L or greater.**

### 4.3 Treatment and follow-up of thyroid dysfunction

Thyrotoxicosis is most often transitory and its treatment depends on the clinical severity and etiological diagnosis. Treatment must be decided on in agreement between the treating endocrinologist and oncologist or specialist. Corticosteroid therapy should be considered only in clinically severe cases of thyrotoxicosis.

Hypothyroidism most often appears as a second phase after thyrotoxicosis induced by silent inflammatory thyroiditis. The decision to treat should take into account the general state of the patient, the presence of comorbidities, especially cardiac comorbidity, as well as the clinical and biochemical severity of the hypothyroidism. The evolution of hypothyroidism after withdrawal of ICPI is not well known. Thyroid hormones are usually necessary on a long-term basis in patients with hypothyroidism following a transitory thyrotoxicosis, but follow-up is not consensual (de Filette *et al.* 2016, Delivanis *et al.* 2017, Haanen *et al.* 2017, Illouz *et al.* 2017, Morganstein *et al.* 2017)

**R4.3a: The treatment strategy for thyroid abnormalities in the course of ICPI for cancer should be agreed between the treating endocrinologist and the clinician prescribing ICPI.**

**R4.3b: In cases of asymptomatic thyrotoxicosis, clinical and hormonal monitoring can be proposed. In the case of symptomatic thyrotoxicosis, we recommend treatment, with beta-blockers in the absence of contraindications.**

**R4.3c: The treatment of ICPI-induced hypothyroidism is based on levothyroxine. This is justified in the case of TSH concentrations superior to 10 miU/L and should be discussed in TSH between 5 and 10 mIU/L (found on two consecutive assays), associated with either clinical symptoms or the presence of anti-TPO antibodies. Levothyroxine can be started at a dose of 1–1.6 µg/kg/day but must be adjusted for age, comorbidities and the patients’ prognosis for survival. Modalities for adjusting the dose are the same as for other cases of hypothyroidism.**

**R4.3d: Thyrotoxicosis is transitory and generally evolves toward hypothyroidism. In the case of hypothyroidism, recovery of normal thyroid function is possible but unpredictable. It is recommended to administer thyroid replacement during the entire duration of ICPI. During treatment with levothyroxine, monitoring is based on TSH assays which need to be performed every 3 months. Progressive withdrawal of levothyroxine is possible at the end of ICPI, with continued clinical and TSH monitoring. The presence of thyroid dysfunction secondary to a first treatment with an ICPI (anti-CTLA-4, anti-PD-1 or anti-PD-L1) is not a contraindication for use of a different ICPI.**

## 5. Induced hypophysitis

**Figure 4:**
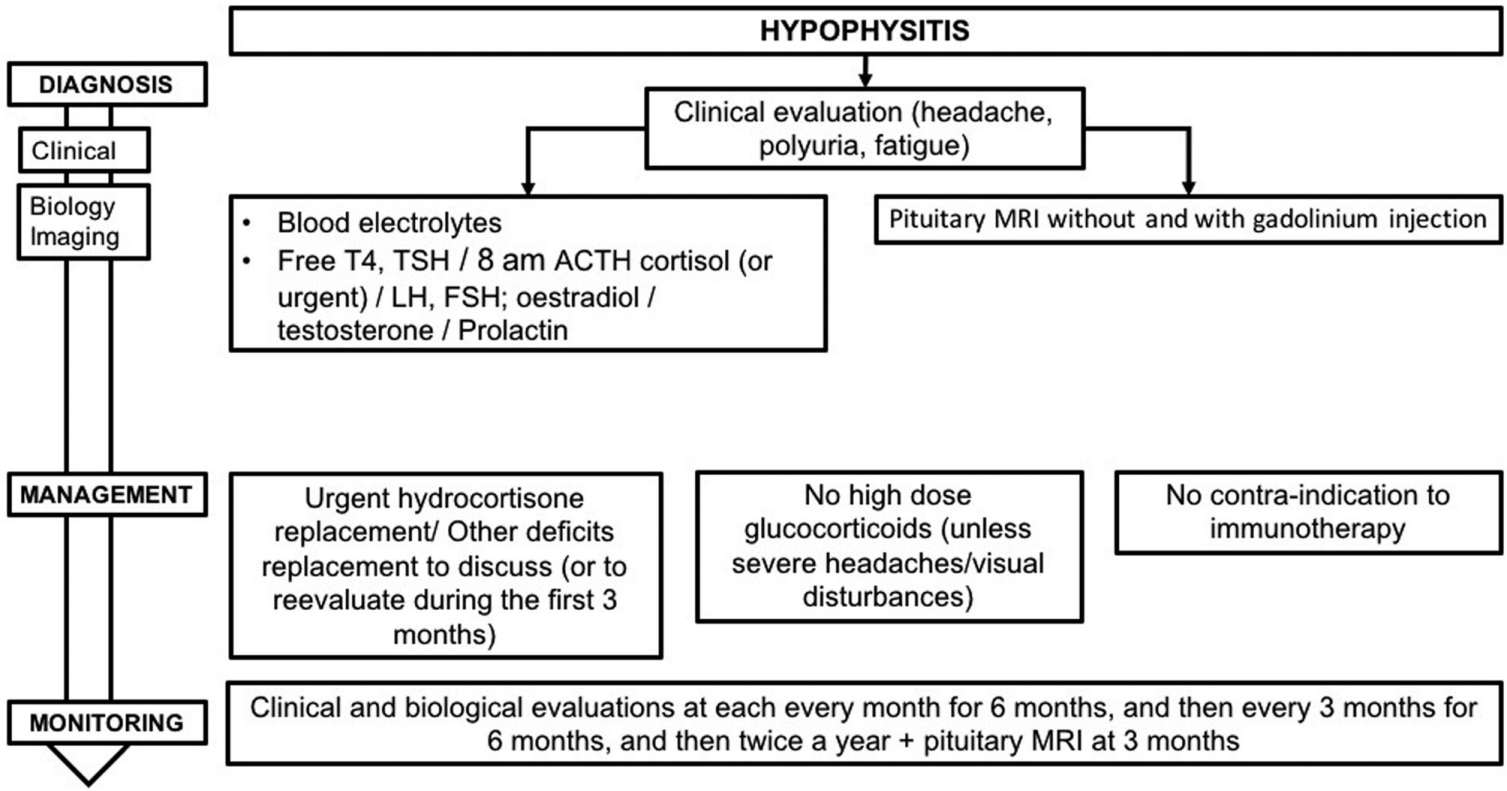
Management and monitoring of ICPI-induced hypophysitis.

Hypophysitis secondary to ICPI appears most often in men over the age of 60 years, with a risk 2–5 times higher than that in women. Its prevalence depends on the treatment used either for monotherapy or in combination (4–20% on ipilimumab, 8% in the combination ipilimumab/nivolumab, 0.6% on nivolumab and 0.7% in pembrolizumab) (Torino *et al.* 2013). The time taken for hypophysitis to appear varies depending on the ICPI used: it can be very early with combined treatments (30 days on average) (Scott *et al.* 2018), between 2 and 3 months (4 weeks up to 19 months) on anti-CTLA-4 (Dillard *et al.* 2010, Weber *et al.* 2012, Albarel *et al.* 2015, Joshi *et al.* 2016, Scott *et al.* 2018), and between 3 and 5 months on anti-PD-1/PD-L1 (Torino *et al.* 2013, Eigentler *et al.* 2016). The pathophysiological mechanisms are not completely understood. In mouse models of hypophysitis, after repeated injections of anti-CTLA-4, lymphocyte infiltration of pituitary tissue as well as the presence of circulating anti-pituitary antibodies has been reported. Similarly, anti-pituitary antibodies have been reported in patients with hypophysitis secondary to anti-CTLA-4 treatment. Injection of anti-CTLA-4 can cause pituitary toxicity by binding to a CTLA-4 antigen that is naturally expressed on pituitary cells (Iwama *et al.* 2014, Faje 2016, Mei *et al.* 2016). Direct binding between ipilimumab and anterior pituitary cells can activate cell-mediated antibody-dependent cytotoxicity (Laurent *et al.* 2013, Romano *et al.* 2015). The involvement of complement activation may also explain the difference in prevalence of hypophysitis induced by different ICPI (Garred *et al.* 1989, Bruhns *et al.* 2009, Briet *et al.* 2018) (Fig. 4).

### 5.1 Diagnosis

Clinical signs tend to be non-specific with the most frequently reported being headaches and profound fatigue. Visual problems or the presence of polyuric–polydipsic syndrome have been reported, though infrequently (Dillard *et al.* 2010, Faje *et al.* 2014, Albarel *et al.* 2015, Faje 2016, Joshi *et al.* 2016, Byun *et al.* 2017). More marked symptoms are found when anti-CTLA-4 and anti-PD-1 are used in combination (Scott *et al.* 2018). Hormonal deficiencies, often multiple at the time of diagnosis, may be associated with hyponatremia in 47–50% of cases, more often in patients who present with cerebral metastases (Faje *et al.* 2014, Albarel *et al.* 2015, Scott *et al.* 2018). Deficiencies in TSH (86–100%), gonadotropin (85–100%) and ACTH (50–73%) are also frequently found. Diabetes insipidus is rare. Pituitary MRI is the most sensitive imaging technique for diagnosis. It variably (30–100%) shows a moderate increase in pituitary volume (with a convex shape), a strong increase in pituitary intensity after injection of gadolinium, which may be heterogeneous, and sometimes enlargement of the pituitary stalk (Albarel *et al.* 2015, Min *et al.* 2015, Scott *et al.* 2018). The changes in MRI can be mild, transitory and sometimes only identifiable when compared to earlier MRI images (Min *et al.* 2015). In this tumoral context, MRI also allows the elimination of other pathologies (metastasis, infectious pathology, apoplexy, pituitary adenoma, infiltrative pathology etc.) (Joshi *et al*. 2016). The diagnosis of hypophysitis is a presumptive diagnosis, since in the absence of indication for surgery there will generally be no final anatomical pathology analysis of the tissue (Caturegli *et al.* 2008, Carmichael 2012).

**R5.1a: The diagnosis of hypophysitis during ICPI should be considered in patients with clinical symptoms evocative of hypophysitis (most frequently headaches and fatigue) and/or hyponatremia and/or pituitary deficiency and/or abnormal pituitary imaging.**

**R5.1b: There is no indication for confirmation of diagnosis of hypophysitis, during ICPI, by histology of a surgical biopsy in the absence of an argument supporting the presence of another pituitary pathology such as a metastasis.**

**R5.1c: In case of suspected hypophysitis, we recommend the following tests:**

- **Blood electrolytes**
- **Assays for hormones, including:**
  - **Free T4 (and TSH due to the risk of thyroid abnormalities on ICPI).**
  - **Plasma cortisol and ACTH (in the context of rare cases described of PAI) at 08:00 h (except in acute situations, cf R6.1b) in the absence of treatment with synthetic glucocorticoids, with dynamic tests depending on these results.**
  - **LH, FSH and estradiol in premenopausal women not taking oral contraceptives for menstrual problems or FSH in post-menopausal women. LH, FSH and total testosterone in men.**
  - **Prolactin levels.**

**It is also important**

- **To look for clinical signs of polyuric–polydipsic syndrome.**
- **To perform a pituitary MRI without and with gadolinium injection, ideally in the acute phase, with the goal of confirming the diagnosis and eliminating differential diagnoses (notably of pituitary metastasis). A normal image on MRI does not exclude the diagnosis.**

In case of abnormal MRI suggestive of hypophysitis, without pituitary deficiency, closer monitoring of hormone levels should be put in place, as symptoms and pituitary deficiencies can appear in a second phase (Faje *et al.* 2014, Min *et al.* 2015, Faje 2016, Scott *et al.* 2018).

**R5.1d: In case of MRI results suggestive of hypophysitis but in the absence of pituitary deficiency, closer monitoring of 08:00 h cortisol levels (weekly during 1 month and then at normal intervals) should be established.**

### 5.2 Treatment

In the acute phase of hypophysitis post ICPI, treatment by high-dose glucocorticoids is not systematically recommended, as neither its efficacy nor lack of harmful effects has been shown. It can be suggested in patients presenting with major headaches, visual aberrations or other auto-immune side effects that justify its use (Lammert *et al.* 2013, Albarel *et al.* 2015, Kumar *et al.* 2017).

**R5.2a: In confirmed cases of hypophysitis, we do not recommend the systematic use of high-dose synthetic glucocorticoids. This treatment may be proposed to treat symptoms such as major headaches that do not respond to normal analgesics and/or in the case of visual disturbances.**

Urgent hydrocortisone intake in the case of ACTH deficiency. Since recovery of the ACTH axis occurs only in exceptional cases, educating the patient and oncologist on adjusting the dose of hydrocortisone, and injecting hydrocortisone hemisuccinate in case of emergency or intercurrent illness, is required (Downey *et al.* 2007, Faje *et al.* 2014, Ryder *et al.* 2014, Albarel *et al.* 2015, Byun *et al.* 2017, Kumar *et al.* 2017). In the case of TSH or gonadotropin deficiency, initiation of treatment is less urgent, recovery of the TSH and gonadotropin axes usually occurs within months following hypophysitis secondary to ICPI (Faje *et al.* 2014, Albarel *et al.* 2015, Min *et al.* 2015, Scott *et al.* 2018).

**R5.2b: In case of suspicion of acute ACTH insufficiency in a patient treated with ICPI, plasma cortisol assay should be urgently carried out, regardless of the time of day, and 100 mg hydrocortisone hemisuccinate should be administered via intravenous, intramuscular or subcutaneous injection, then continuous perfusion of 100 mg delivered over 24 h (as for a non-ICPI-related ACTH insufficiency). This should be initiated without waiting for results of plasma cortisol assay. After improvement in clinical symptoms and biochemical parameters, treatment should be continued by oral hydrocortisone at a dose of 60 mg/24 h, in three administrations, to be reduced progressively until the replacement dose is reached.**

**R5.2c: In case of chronic ACTH deficiency in patients treated with ICPI, the daily dose of hydrocortisone should be 15–20 mg/day (taken in 2 or 3 administrations per day), to be adjusted depending on clinical parameters. The patient needs to be followed-up by an endocrinologist to ensure they are educated regarding their therapy, especially in terms of dose increase in stressful situations (such as infection or trauma) or the need for lifetime replacement. How to adjust the hydrocortisone dose in case of an acute medical event should also be explained to the referring oncologist.**

**R5.2d: In case of TSH deficiency, treatment with levothyroxine should be considered on a case-by-case basis, depending on the severity of the deficiency, clinical tolerance and/or the clinical and biochemical evolution seen after thyroid tests carried out at 1 month.**

**R5.2e: In case of gonadotropin deficiency, replacement should be considered depending on the evolution of the gonadotropin deficiency in the first 3 months of monitoring and in the absence of oncological contraindication.**

**R5.2f: Confirmed cases of diabetes insipidus should be systematically treated.**

**R5.2g: Given the oncological context, no replacement therapy should be considered in the case of growth hormone deficiency.**

In cases of hypophysitis, ICPI can be continued, after the management of acute hormonal deficiencies (Albarel *et al.* 2015, Min *et al.* 2015, Faje 2016, Joshi *et al.* 2016, Kumar *et al.* 2017). Withdrawal of ICPI has been reported to have no effect on the natural history of hypophysitis (Min *et al.* 2015). There is currently no published data on the risk of hypophysitis in patients with a history of pituitary pathology prior to ICPI. In these patients, close examination of hormone equilibrium obtained prior to ICPI is needed and can lead to a readjustment of dose for replacement therapies.

**R5.2h: Hypophysitis is not a contraindication for ICPI, which can be delayed in the acute phase of hypophysitis. The presence of hypophysitis secondary to a first ICPI (anti-CTLA-4, anti-PD-1 or anti-PD-L1) does not contraindicate the use of another ICPI. A history of pituitary pathology does not contraindicate treatment by ICPI. Adjustment of replacement therapy may be necessary.**

### 5.3 Follow-up

Recovery from pituitary deficiencies is variable and new deficiencies can appear secondarily. ACTH deficiency persists in 86–100% of cases, while 13–36% of patients continue to have TSH deficiency and 13–53% a gonadotropin deficiency (Albarel *et al.* 2015, Min *et al.* 2015). Recovery from TSH and gonadotropin deficiencies occurs after 10–15 weeks (Min *et al.* 2015). Pituitary hypertrophy regresses in 73% of cases (Min *et al.* 2015). Given the principal differential diagnosis represented by pituitary metastasis, new imaging by MRI should be carried out 3 months after the diagnosis.

**R5.3: In patients presenting with hypophysitis, we suggest clinical and hormonal monitoring (anterior pituitary hormone tests to check for new deficiencies and to adjust replacement therapy) at each appointment for 6 months, then at three-monthly specialist consultations for 6 months and bi-annually thereafter. In view of the possibility that there may be functional recovery of hormonal axes and depending on the clinical state of the patient, cessation of replacement treatments could be considered while continuing with specialist follow-up. We recommend a new pituitary MRI at 3 months to eliminate the differential diagnosis of pituitary metastasis and to assess the evolution of pituitary inflammation.**

## 6. Induced PAI: diagnosis and management

**Figure 5:**
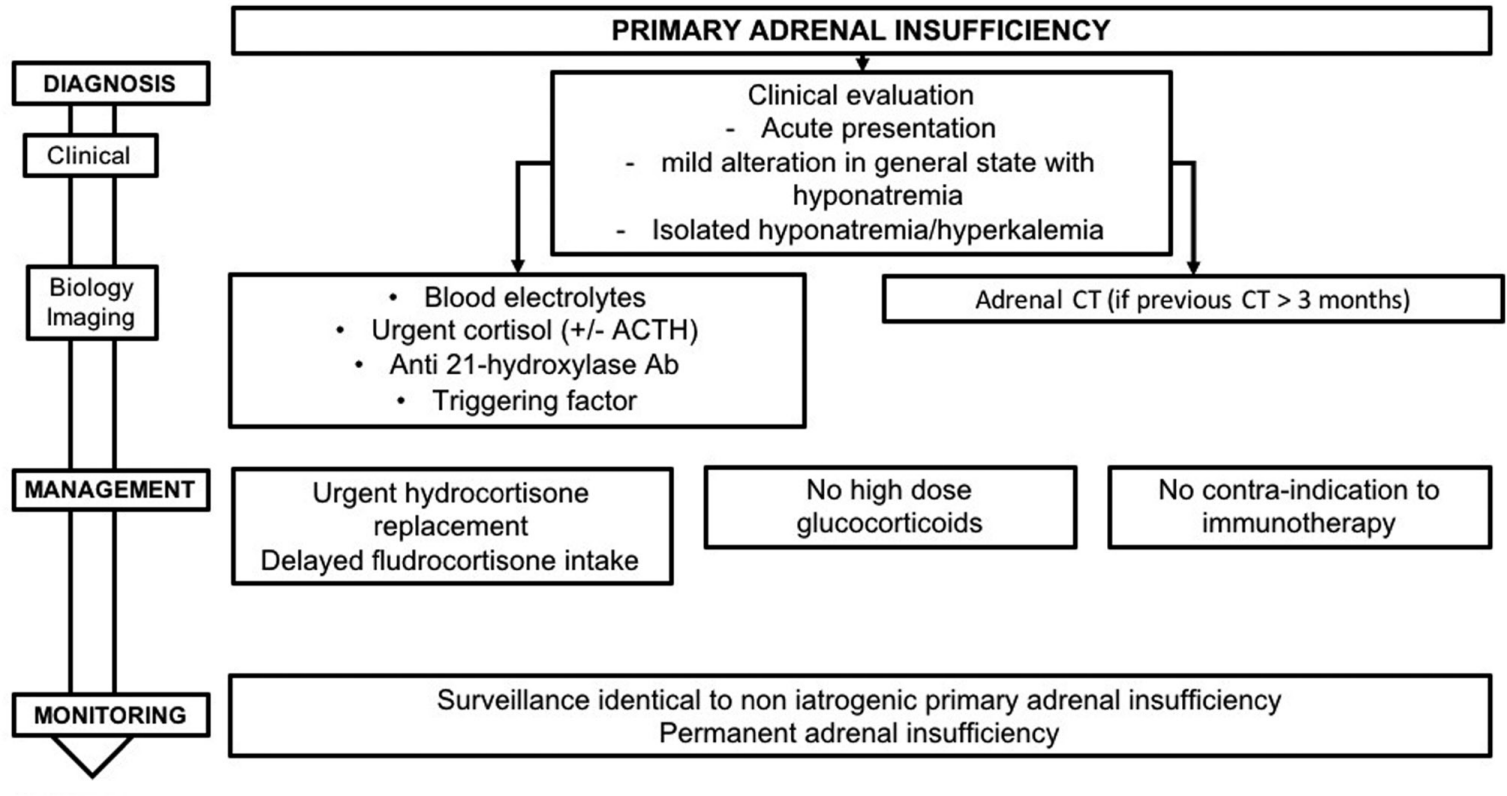
Management and monitoring of ICPI-induced primary adrenal insufficiency.

Published series of cancer patients, describe a less than 1% incidence of PAI in the case of monotherapy and 4–8% in the case of combined ICPI (Barroso-Sousa *et al.* 2017, Byun *et al.* 2017, Cukier *et al.* 2017). However, as the etiology (primary or secondary) of the adrenal insufficiency was not specified, these figures need to be interpreted with caution. To date, there are only six cases sufficiently well documented to confirm the existence of PAI. Conversely, ACTH insufficiency has been more frequently described (Yang *et al.* 2007, Weber *et al.* 2008, Hodi *et al.* 2010, Hersh *et al.* 2011, Madan *et al.* 2012, Wolchok *et al.* 2013, Kwon *et al.* 2014, Ryder *et al.* 2014, Postow *et al.* 2015, Rizvi *et al.* 2015, Herbst *et al.* 2016, Gulley *et al.* 2017). Treatments that have already resulted in confirmed ICPI-induced PAI include ipilimumab, pembrolizumab and nivolumab. The underlying pathophysiological mechanism for this is unknown. Anti-adrenal antibodies have been detected in two patients (Paepegaey *et al.* 2017, Hescot *et al.* 2018). Some studies have reported a morphological appearance of adrenal inflammation (Min & Ibrahim 2013, Bacanovic *et al.* 2015). Fluorodeoxyglucose-PET scanning showed uniform hypermetabolism of both adrenals (Bacanovic *et al.* 2015, Trainer *et al.* 2016). Adrenal atrophy has also been described in one patient (Hescot *et al.* 2018). The definitive character of the adrenal insufficiency described in these clinical cases is consistent with an auto-immune destruction of the adrenals induced by ICPI (Haissaguerre *et al.* 2018) (Fig. 5).

### 6.1 Positive diagnosis

There are no specific clinical signs for PAI induced by ICPI. Two clinical situations have been described:

- A typical acute presentation (Paepegaey *et al.* 2017).
- Or a sub-acute picture that is more progressive (Trainer *et al.* 2016). Some patients can also present with isolated hyponatremia.

The median time for appearance of PAI seems to be quite variable, from 2.5 to 5 months depending on the drug used (Cukier *et al.* 2017). PAI can also appear later, after ICPI withdrawal, as has been described for pembrolizumab (Paepegaey *et al.* 2017).

**R6.1a: Diagnosis of PAI in a patient undergoing ICPI must be considered in the case of:**

- **A clinical picture of acute symptoms suggestive of PAI (fatigue, weight-loss, dehydration, hypotension, fever, abdominal pain, nausea, vomiting, diarrhea, muscular pain and cramps).**
- **A clinical picture of mild alteration in general state which includes hyponatremia.**
- **More rarely, isolated hyponatremia or hyperkalemia.**

**R6.1b: In an emergency, in case of suspicion of acute adrenal insufficiency in a patient treated with ICPI, we recommend:**

- **Immediate blood sample be taken for plasma cortisol assay and, if possible, ACTH, regardless of time of day.**
- **Begin hydrocortisone supplementation without waiting for test results.**

**The diagnosis of adrenal insufficiency can be excluded if plasma cortisol is >500 nmol/L regardless of time of day. This threshold needs to be considered with care and should be discussed in relation to the assay kits that are employed.**

**R6.1c: In non-urgent situations, diagnosis of PAI is confirmed by 08:00 h plasma cortisol being <138 nmol/L (5 µg/dL) and assay showing plasma ACTH being elevated. If 08:00 h cortisol is between 138 and 500 nmol/L (5–18 µg/dL), a stimulation test, such as the Synacthen 250 µg test, should be performed as secondary intention for diagnosing ‘latent’ adrenal insufficiency. If cortisol levels in the course of the test are <500 nmol/L (18 µg/dL), diagnosis of adrenal insufficiency is confirmed. These thresholds need to considered carefully and discussed in relation to the assay kits employed.**

### 6.2 Etiological diagnosis

The primary or secondary etiology of adrenal insufficiency is determined by assay for ACTH. In the case of PAI, assay for anti-21 hydroxylase antibodies should be carried out to examine the possibility of an auto-immune cause. If abdominal imaging results are older than 3 months (performed as part of cancer follow-up), adrenal CT should be performed to look for variations in adrenal morphology suggestive of adrenal inflammation or adrenal atrophy and to eliminate the differential diagnosis of bilateral adrenal metastases or tuberculosis.

**R6.2: In case of diagnosis of PAI in a patient treated with ICPI, we recommend:**

- **Testing for the presence of anti-21 hydroxylase antibodies.**
- **Performing a (non-urgent) adrenal CT scan, where abdominal imaging dates from more than 3 months, to eliminate other etiologies (adrenal metastases, infection, bilateral necrotic hemorrhage of the adrenals or granulomatosis).**

### 6.3 Treatment

In cases of acute PAI, patient care must be initiated urgently. Since recovery is unusual, education of both the patient and the oncologist is necessary regarding adjustment of hydrocortisone dose and injection of hydrocortisone hemisuccinate.

**R6.3a: In case of acute adrenal insufficiency in a patient undergoing ICPI, we recommend intravenous injection (otherwise intramuscular or subcutaneous injection) of 100 mg hydrocortisone hemisuccinate and then to initiate continuous infusion of 100 mg hydrocortisone hemisuccinate over 24 h and rehydration therapy (as for acute adrenal insufficiency not linked to ICPI). When clinical and biochemical parameters have improved, continuation of treatment using oral administration of 60 mg/24 h hydrocortisone should be established. In the absence of acute pathology, the dose can be reduced thereafter to 15–30 mg/24 h. Treatment using fludrocortisone could then be started, at 50 µg/day, to be adjusted later by an endocrinologist.**

**R6.3b: In case of acute adrenal insufficiency, ICPI can be discontinued, but should in no case be definitively contraindicated. ICPI can be reintroduced at normal dose when correct replacement therapy has been established using hydrocortisone and the patient is clinically and biochemically (blood electrolytes) stabilized.**

Long-term treatment of PAI is the same as that recommended for management of Addison’s disease (Castinetti *et al.* 2017). It includes hydrocortisone (dose required being approximately 9.9 + 2.2 mg/m^2^/day, thus, a total of 20–30 mg, taken as two or three doses per day) and fludrocortisone (average dose 100 µg/day).

**R6.3c: In case of known PAI in a patient treated with ICPI, the daily dose of hydrocortisone is 15–30 mg/day, to be adjusted as a function of clinical and biochemical parameters and of the general state of the patient. At these doses, replacement is not immunosuppressive. Supplementation with fludrocortisone can be adjusted as a function of blood pressure, serum potassium and if available, of plasma renin. Patient follow-up should be by an endocrinologist and therapeutic education should be provided, aimed at preventing acute episodes. Equally, the manner in which to adjust hydrocortisone dose, in case of an acute medical event, needs to be explained to the referring oncologist.**

### 6.4 Follow-up

In view of the rarity of PAI, there is insufficient data to recommend systematic 08:00 h plasma cortisol assays before or during ICPI, or for systematic testing for anti-21 hydroxylase antibodies. The few published studies suggest that ICPI-induced PAI can appear during or after the cessation of ICPI.

**R6.4a: Considering the rarity of the condition, there is no indication for systematic screening for PAI, before or during ICPI, except in the case of clinical signs evocative of PAI.**

PAI in such cases appears to be permanent, though the number of cases reported is small and the maximal follow-up being 1 year (Trainer *et al.* 2016).

**R6.4b: The available data suggest that replacement therapy must be open-ended.**

## 7. Induced diabetes: diagnosis and management

Isolated cases of ICPI-induced diabetes are reported in the literature, as well as some published series consisting of a few patients. Two publications, combining several clinical trials, report an overall incidence of diabetes of 0.4% (Byun *et al.* 2017, Sznol *et al.* 2017). A recent study reported a prevalence of 0.9% among 2960 patients treated by ICPI (Stamatouli *et al.* 2018). Of note, diabetes has been described in the case of patients treated with anti-PD-1/PD-L1, but not with anti-CTLA-4.

**Figure 6:**
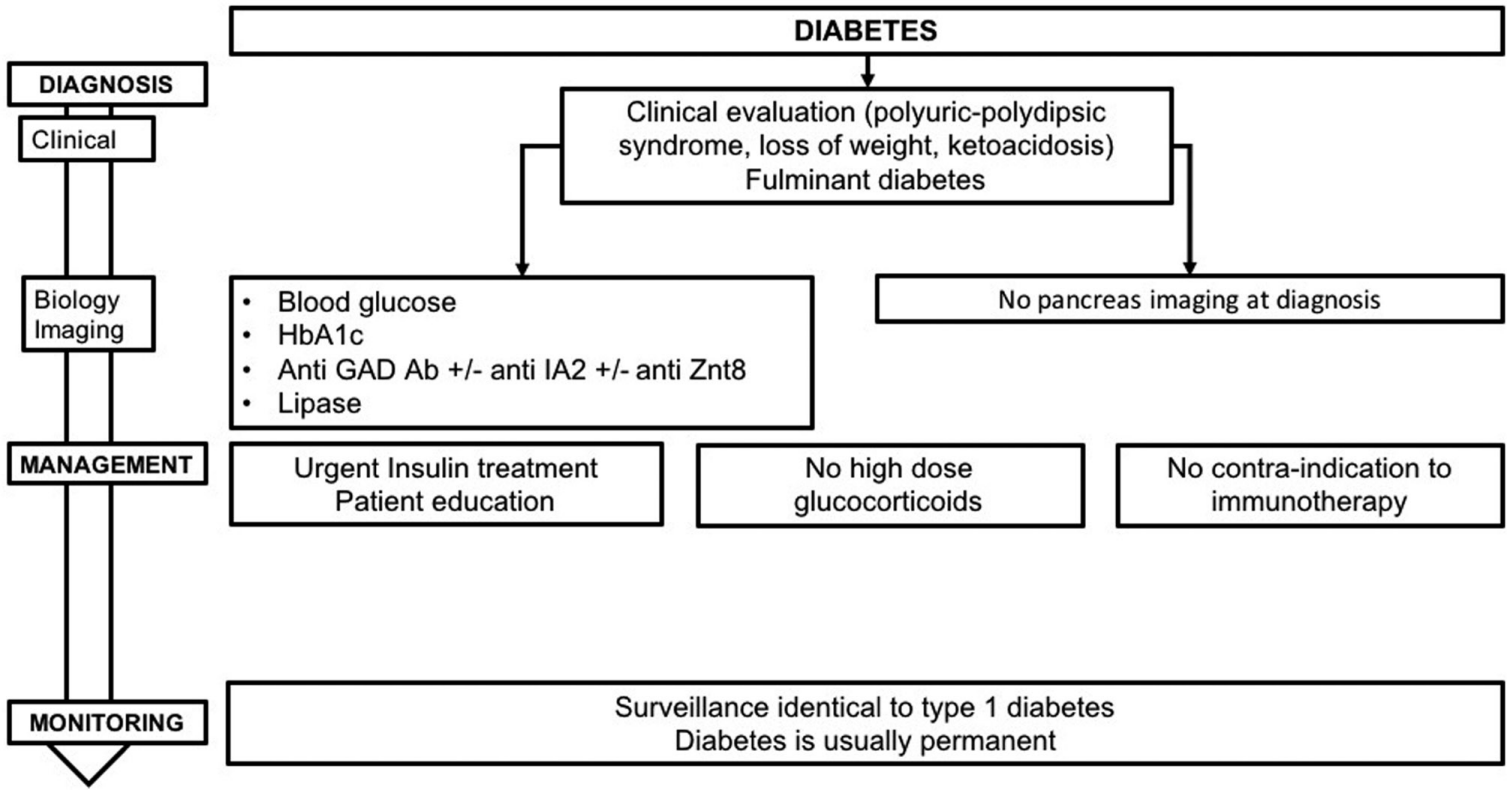
Management and monitoring of ICPI-induced diabetes.

In terms of pathophysiology, PD-L1 is expressed in pancreatic islet cells and the interaction PD-1/PD-L1 seems to play a protective role against auto-immune diabetes by inhibiting the activation of auto-reactive T lymphocytes (Keir *et al.* 2006). Evidence that PD-1 inhibitors are implicated in the development of auto-immune diabetes has been provided by studies in NOD (non-obese diabetic) mice, a mouse model frequently used for studying auto-immune diabetes (Ansari *et al.* 2003). Injection of anti-PD-1 or anti-PD-L1 caused the development of diabetes in NOD mice, the appearance being, on average, a few days after antibody administration (Ansari *et al.* 2003). In this same study, no mice developed diabetes in response to administration of anti-CTLA-4 antibodies. Histological analysis of the pancreas in these mice showed massive destructive insulitis in NOD mice treated with anti-PD-1 or anti-PD-L1 antibodies, while age-matched control mice showed only minimal inflammation of pancreatic islets. No link was shown between the appearance of diabetes and the presence of anti-insulin auto-antibodies. Finally, it has also been hypothesized that a particular intestinal microbiome could predispose to undesirable auto-immune effects and thus represents a risk factor. Preliminary preclinical and clinical data suggest an association between particular strains of bacteria and the efficacy of immunotherapies (Sivan *et al.* 2015, Vétizou *et al.* 2015, Smati *et al.* 2018) (Fig. 6).

### 7.1 Diagnosis

Classical symptoms of insulinopenia characteristic of type 1 diabetes (DT1) can be identified, such as polyuria, polydipsia, loss of weight or fatigue (Sznol *et al.* 2017). Diagnosis is confirmed biochemically by the presence of frank hyperglycemia. In the most severe forms, which are also the majority (fulminant diabetes), the clinical picture is associated with ketoacidosis. In the recent largest reported study (27 patients), ketoacidosis was reported in 57% cases; 42% of the patients had evidence of pancreatitis in the peridiagnosis period; mean blood glucose was 6.53 g/L, mean HbA1c was 7.95% and the C-peptide was very low (Stamatouli *et al.* 2018). Exocrine pancreatic function has not been systematically explored, though it has been recently shown in one case of diabetes induced by nivolumab (anti-PD-1), with both a bi-hormonal deficiency (insulin and glucagon) and an exocrine pancreas insufficiency (asymptomatic reduction in fecal elastase) (Marchand *et al.* 2018). Diabetes has been diagnosed after a mean time of 20 weeks after the initiation of treatment with PD-1 or PD-L1 inhibitors (the most delayed cases appearing 54 months after treatment). Antibodies are present in half of the reported patients, and among these anti-GAD antibodies have been consistently found. Of note, there was a predominance of HLA-DR4 that was reported in 76% of the patients with ICPI-induced diabetes in the largest reported study (Stamatouli *et al.* 2018).

**R7.1a: In patients treated with anti-PD-1 or anti-PD-L1, appearance of polyuric–polydipsic syndrome, loss of weight or clinical signs evoking ketoacidosis should lead to immediate testing of blood glucose. Measurement of HbA1c should be carried out in cases of pathological hyperglycemia. As first intention, testing for anti-GAD antibodies should be performed and if these are absent, testing for anti-IA2 and anti-ZnT8 antibodies should be carried out. Tests for pancreatic lipase should also be performed in cases of clinical presentation of fulminant diabetes. Imaging of the pancreas is not indicated at diagnosis.**

Taking into account the often sudden nature of the diagnosis (‘fulminant-like’ diabetes) it is important to educate patients on recognition of the first diabetic symptoms that may appear (polyuria–polydipsia, vomiting and abdominal pains).

**R7.1b: We recommend educating patients who require anti-PD-1 or PD-L1 treatment on the first signs of diabetes (polyuric–polydipsic syndrome, weight-loss) or ketoacidosis (vomiting, digestive troubles).**

### 7.2 Treatment

All described patients have been treated using multiple injections of insulin. In follow-up, the objective is to maintain HbA1c lower than 8.0%. In the absence of published data supporting its efficacy, corticosteroid therapy is not indicated. Patients can receive ICPI in parallel with initiation of insulin therapy and management of their diabetes, except in severe cases where ICPI could be delayed by a few days.

**R7.2a: Since treatment with ICPI using anti-PD-1 or PD-L1 can lead to presentation of fulminant symptoms of diabetes with major insulinopenia, we recommend urgently initiating insulin treatment via multiple injections as first line treatment, and patient management and diabetes education in a specialized service. The aim is to maintain HbA1c at <8.0%. There are no alternative treatments for ICPI-induced diabetes.**

**R7.2b: The development of diabetes during ICPI with anti-PD-1 or PD-L1 does not contraindicate continuing ICPI. Where the situation is severe, ICPI can be delayed for a few days.**

### 7.3 Follow-up

There is little published data concerning patients who are diabetic prior to starting ICPI. Nevertheless, we recommend increased surveillance of capillary glycemia after commencing ICPI. There is no published data at present suggesting a possible remission of diabetes after withdrawal of ICPI.

**R7.3a: In case of pre-existing diabetes in patients treated with anti-PD-1 or anti-PD-L1, self-monitoring of blood glucose should be proposed or reinforced in those patients who were already undertaking this. We do not recommend monitoring of glycemia in patients being treated with anti-CTLA-4 alone.**

**R7.3b: Since the induced diabetes is generally permanent, we recommend continuing treatment and follow-up after the cessation of ICPI.**

## Conclusions

In conclusion, the appearance of an endocrinopathy during anti-cancer therapy necessitates coordinated patient management by the endocrinologist and oncologist. The risk of endocrinopathy justifies patient education but also the training of treating oncologists, thus helping to orient them toward early diagnosis. Overall, the two fundamental steps are the commencement and adjustment of replacement therapy. The procedures then for the eventual cessation of this treatment must, in all cases, be discussed jointly by the various medical specialists involved in caring for the patient. Lastly, the classical instructions in the CTCAE, relating to ICPI-induced endocrinopathies, need to be followed with caution to avoid the unjustified stopping of an effective anti-tumor treatment to the detriment of patient survival (Castinetti *et al.* 2018).

## Declaration of interest

The authors declare that there is no conflict of interest that could be perceived as prejudicing the impartiality of this guideline.

## Funding

This work did not receive any specific grant from any funding agency in the public, commercial or not-for-profit sector.
